# The use of low-dose naltrexone (LDN) as a novel anti-inflammatory treatment for chronic pain

**DOI:** 10.1007/s10067-014-2517-2

**Published:** 2014-02-15

**Authors:** Jarred Younger, Luke Parkitny, David McLain

**Affiliations:** 1 Stanford University, Stanford, CA USA; 2 Department of Anesthesia, Pain and Perioperative Medicine, Stanford University, 1070 Arastradero Road, Suite 200, Palo Alto, CA 94304 USA; 3 McLain Medical Associates, Birmingham, AL USA; 4 Department of Anesthesia, Pain and Perioperative Medicine, Stanford University, 1070 Arastradero Road, Room 286, Palo Alto, CA 94304 USA

**Keywords:** Anti-inflammatory, Chronic pain, Fibromyalgia, Glial cell modulators, Low-dose naltrexone, Microglia

## Abstract

Low-dose naltrexone (LDN) has been demonstrated to reduce symptom severity in conditions such as fibromyalgia, Crohn’s disease, multiple sclerosis, and complex regional pain syndrome. We review the evidence that LDN may operate as a novel anti-inflammatory agent in the central nervous system, via action on microglial cells. These effects may be unique to low dosages of naltrexone and appear to be entirely independent from naltrexone’s better-known activity on opioid receptors. As a daily oral therapy, LDN is inexpensive and well-tolerated. Despite initial promise of efficacy, the use of LDN for chronic disorders is still highly experimental. Published trials have low sample sizes, and few replications have been performed. We cover the typical usage of LDN in clinical trials, caveats to using the medication, and recommendations for future research and clinical work. LDN may represent one of the first *glial cell modulators* to be used for the management of chronic pain disorders.

## Introduction

In this review, we will discuss the concept of using *low-dose naltrexone* (LDN) as a novel anti-inflammatory treatment for chronic pain conditions that are suspected to be associated with inflammatory processes. Within a specific dosage window, opioid antagonists such as naltrexone can exert a “paradoxical” analgesic effect [1]. We will further present the rationale for considering LDN as a primary example of a relatively new class of therapeutic agents called *glial cell modulators*. This review is intended for clinicians who are seeking additional information about the background, theory, mechanism of action, and research use of LDN. We will be focusing this discussion on LDN as a monotherapy for chronic pain. The closely related concept of ultralow-dose naltrexone involves the use of microgram, nanogram, and picogram dosages of naltrexone co-administered with opioid analgesics [2]. The approach is used to both increase the efficacy of opioid analgesia therapy and reduce some adverse side effects. Ultralow-dose naltrexone has been covered extensively in previous reviews [3] and will not be discussed here.

## Background

Naltrexone was synthesized in 1963 as an orally active competitive opioid receptor antagonist [4]. Naltrexone is structurally and functionally similar to the opioid antagonist naloxone, but it has greater oral bioavailability and a longer biologic half-life [5]. Naltrexone HCl was approved by FDA in 1984 for the treatment of opioid addiction. The typical daily dosage for opioid addiction is 50.0–100.0 mg daily, and 50.0-mg tablets are available commercially. A more complete review of the early history of naltrexone can be found elsewhere [6].

LDN refers to daily dosages of naltrexone that are approximately 1/10th of the typical opioid addiction treatment dosage. In most published research, the daily dosage is 4.5 mg, though the dosage can vary a few milligrams below or above that common value [7–9]. At the low dosage level, naltrexone exhibits paradoxical properties, including analgesia and anti-inflammatory actions, which have not been reported at larger dosages. LDN was reported to have interesting physiological properties (primarily enhancement of endogenous opioid production) in the 1980s [6], and the treatment approach was reported to be used clinically since the mid-1980s [10]. Basic science work examining the use of opioid antagonists for treating disease states did not start to appear until the late 1980s [11], and the first published LDN trial in humans was presented in 2007 [12]. Since that time, LDN has been studied in a small number of labs and has been slowly gaining attention as a possible treatment for some chronic medical conditions.

## Use of LDN in chronic pain

**Fig. 1:**
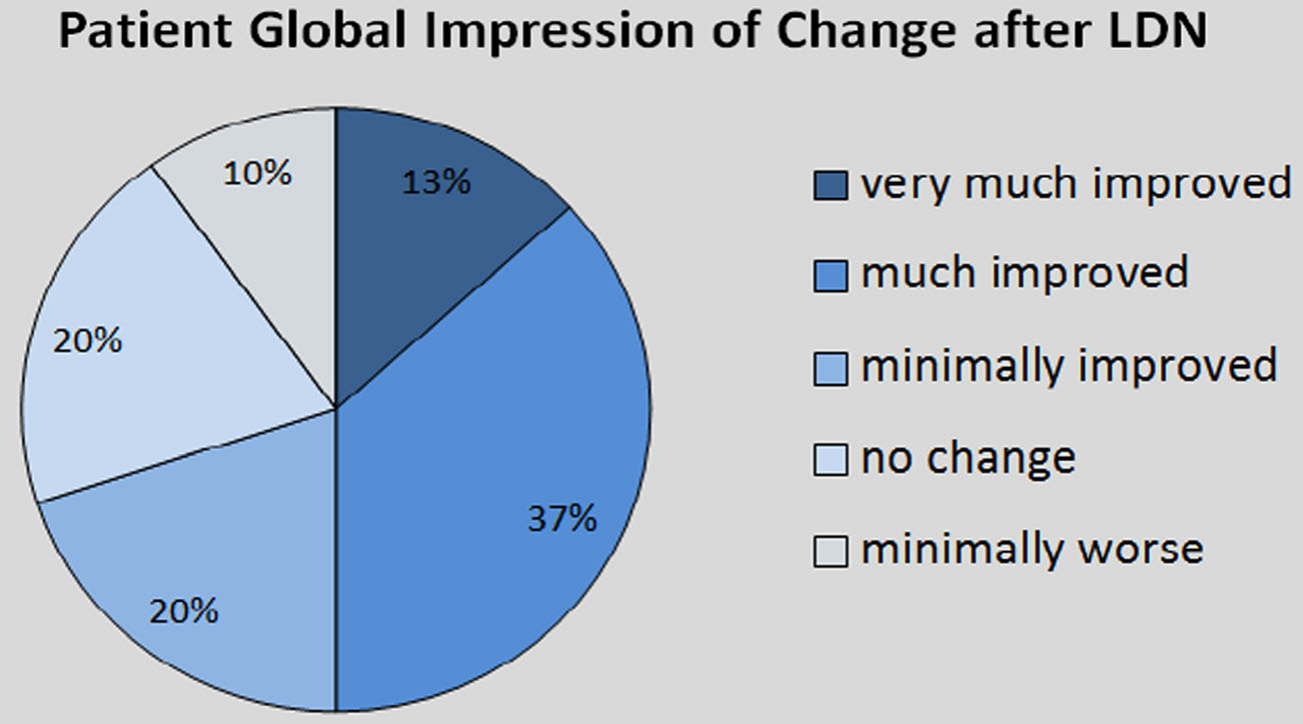
Fibromyalgia participants’ (*N* = 29) self-reported improvement in symptoms after daily LDN treatment. The figure uses data from an earlier clinical trial [9] and has not been previously published

LDN has been tested experimentally in a small number of chronic pain conditions. One such condition is fibromyalgia (FM). FM is a chronic pain disorder that is characterized by diffuse musculoskeletal pain and sensitivity to mechanical stimulation as well as profound fatigue, cognitive disruption, and sleep difficulty. Although FM does not respond to common anti-inflammatories and does not seem to be an inflammatory disorder in the classic sense [13], inflammatory processes may still be involved [14]. We have shown in two separate, small clinical trials that LDN may be an effective treatment for FM. In both trials, LDN was administered at 4.5 mg daily, once at night before bedtime. In the first crossover trial, published in 2009 [15], LDN reduced fibromyalgia pain significantly greater than placebo in 6 out of the 10 women. While the pilot study was encouraging, it had limitations such as a single-blind design. To help validate the findings, a second study in 30 women with fibromyalgia was conducted [9]. In that double-blind, crossover, counterbalanced study, 57 % of the participants were observed to exhibit a significant (1/3) reduction of pain during LDN. At the end of the LDN treatment, half of the participants reported feeling “much improved” or “very much improved” from LDN (Fig. 1). Together, these two studies suggest that LDN is superior to placebo in reducing the pain associated with fibromyalgia.

## Evidence for a novel central anti-inflammatory action of naltrexone

While preliminary evidence exists for the efficacy of LDN, it is critical that we better understand the mechanism of clinical action. This information would allow researchers to develop even more effective treatments for fibromyalgia and other pain disorders. We now present three pieces of evidence to support the argument that LDN may be a useful therapeutic agent in pain conditions that involve ongoing inflammation. First, we will discuss in vivo and in vitro basic scientific evidence of naltrexone’s anti-inflammatory effects. Second, we will identify a relationship between LDN and baseline inflammation. Third, we will mention other inflammatory conditions in which LDN has demonstrated clinical efficacy.

### Anti-inflammatory effects of LDN in vivo and in vitro

In describing LDN’s clinical utility, it is important to understand the dual physiologic mechanisms of naltrexone and other opioid antagonists. Most clinicians are familiar with naltrexone as a potent and nonselective opioid receptor antagonist and treatment for opioid addiction. Naltrexone, at typical dosages, significantly blocks activity at mu- and delta-opioid receptors as well as (to a lesser extent) kappa-opioid receptors [16]. Because beta-endorphin activity at mu-opioid receptors is associated with endogenous analgesic processes, it may seem counterintuitive to administer naltrexone to individuals with chronic pain, as we might expect the medication to reduce analgesia produced by beneficial endogenous opioid activity.

Naltrexone, however, exerts its effects on humans via at least two distinct receptor mechanisms. In addition to the antagonist effect on mu-opioid and other opioid receptors, naltrexone simultaneously has an antagonist effect on non-opioid receptors (Toll-like receptor 4 or TLR4) that are found on macrophages such as microglia [17]. It is via the non-opioid antagonist path that LDN is thought to exert its anti-inflammatory effects. Microglia are central nervous system immune cells that are activated by a wide range of triggers [18]. Once activated, microglia produce inflammatory and excitatory factors that can cause sickness behaviors such as pain sensitivity, fatigue, cognitive disruption, sleep disorders, mood disorders, and general malaise [19]. When chronically activated, the resulting proinflammatory cascade may become neurotoxic, causing several deleterious effects [20]. Given the wide variety of inflammatory factors produced by activated microglia (e.g., proinflammatory cytokines, substance P, nitric oxide, and excitatory amino acids) [21], a range of symptoms and medical outcomes could share the pathophysiological mechanism of central inflammation. Conditions such as fibromyalgia may involve chronic glial cell activation and subsequent production of proinflammatory factors. The hypothesis is indirectly and partially supported by the high degree of symptomatic overlap between fibromyalgia and cytokine-induced sickness behaviors.

Both naloxone and naltrexone have been demonstrated to exert neuroprotective and analgesic effects [22]. The neuroprotective action appears to result when microglia activation in the brain and spinal cord is inhibited [23]. By suppressing microglia activation, naloxone reduces the production of reactive oxygen species and other potentially neuroexcitatory and neurotoxic chemicals [24]. The anti-inflammatory effect of opioid antagonists may also extend to the periphery, as evidenced by suppressed TNF-alpha, IL-6, MCP-1, and other inflammatory agents in peripheral macrophages [25]. It should be noted that most animal work has used naloxone, while most human work has used naltrexone (because of its higher oral availability). We cannot discount the possibility that findings from one compound would imperfectly translate to the other.

The hypothesis that naltrexone and naloxone operate via glial cells to exert their beneficial actions is supported by work with dextro-naltrexone. Dextro-naltrexone is a stereoisomer of naltrexone which is active at microglia receptors but has no activity on opioid receptors [26]. Dextro-naltrexone possesses analgesic and neuroprotective properties [27]. Therefore, the analgesic, anti-inflammatory, and neuroprotective effects of naltrexone do not appear to be dependent on opioid receptors.

The majority of work to date has focused on naloxone/naltrexone’s action on microglia TLR4 (e.g., [28]). However, it should be mentioned that the data do not perfectly fit a TLR4 hypothesis [29], and other targets have been proposed, including astrocytes [30] and NADPH oxidase 2 [31]. Other sites of action, including the opioid growth factor receptor (OGFr) [32], are being discovered, raising even more potential mechanisms of action. Given the multiple and varied sites where naltrexone exhibits significant pharmacologic activity, it will be difficult to determine with certainty the paths that are critical for the clinically beneficial effects. This area of research is being vigorously pursued by multiple laboratories.

### Association with general markers of inflammation

As clinical research of LDN is still in its infancy, we do not have studies in humans that parallel the work performed in animal models. However, some indirect evidence supports the concept of LDN as a novel anti-inflammatory. In the initial pilot study of LDN in fibromyalgia [15], baseline erythrocyte sedimentation rate (ESR) was a significant predictor of clinical response to LDN. ESR is a commonly employed clinical test that is sensitive to both chronic and acute inflammatory processes [33]. In our study, individuals with greater ESR at baseline experienced a greater drop in pain when taking LDN, despite that fact that FM is not considered to be a classic inflammatory disorder, and ESR values were in the normal to high-normal range.

**Fig. 2:**
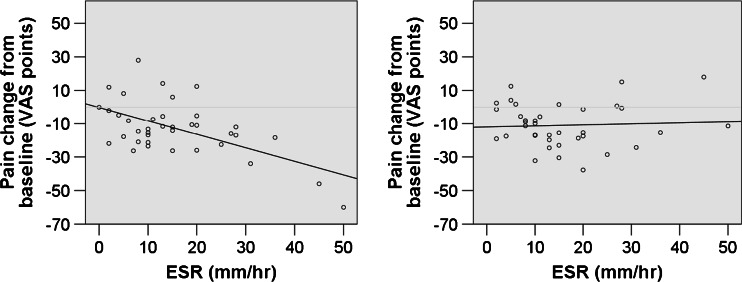
Relationship between baseline erythrocyte sedimentation rate (ESR) and change in pain during administration of LDN (*left pane*) and placebo (*right pane*). The figure uses data from earlier clinical trials [9, 15] and has not been previously published

We have now collected more data on the relationship between baseline ESR and LDN (38 individuals with fibromyalgia in total). Aggregating across studies (Fig. 2), we see that fibromyalgia patients with greater ESR levels at baseline tend to have greater pain reduction when taking LDN (left pane; *r* = 0.58, *p* = 0.0001). In contrast, there is no association between baseline ESR and pain reduction during placebo administration (right pane; *r* = 0.06, *p* = 0.744). Each participant received both LDN and placebo in a blinded fashion. The difference in correlations is significant (*z* = 2.52, *p* = 0.012), suggesting that the clinical effect of LDN may be physiologically associated with the reduction of inflammation. Unfortunately, as we collected ESR only as a screening blood test (to exclude major inflammatory disease), we did not measure ESR at the end of the LDN condition and therefore cannot determine if LDN responders had a significant decrease in their ESR.

We also note that the etiology of FM is controversial, and there is no consensus on pathophysiological mechanisms. FM is not likely to be an inflammatory disorder in the traditional sense but rather a central immune disorder associated with an amplification of pain [13] that involves at least a low level of peripheral cytokine expression (e.g., [34, 35]). The results presented here should be interpreted with caution until replicated in a larger sample. If supported in future research, however, the observed relationship between ESR and LDN response raises the intriguing possibility that other chronic conditions characterized by high ESR may also benefit from LDN therapy.

### LDN has efficacy in treating known inflammatory disorders

A third piece of evidence that suggests LDN may have anti-inflammatory properties in humans is found in the nature of the chronic conditions that appear to respond to LDN treatment. The condition with the most scientific support for LDN efficacy is Crohn’s disease (CD) [7, 12, 36]. CD is an inflammatory bowel disease that exerts gastrointestinal tract and systemic effects. LDN has been reported to reduce not only self-reported pain in that condition but also objective markers of inflammation and disease severity (including the severity scores from endoscopic evaluation) [7, 12, 36]. The response rate of LDN in Crohn’s disease may be even higher than that seen in fibromyalgia, with over 80 % of the study participants exhibiting significant improvement [7, 12].

Naltrexone has also shown some promise in improving disease severity in multiple sclerosis [8], an inflammatory, demyelinating condition of the central nervous system. The evidence of LDN efficacy is not as robust as in the previously mentioned conditions. There is some evidence of reduced spasticity and improved mental health, but many clinical endpoints fail to show difference from placebo, and one study [37] did not find improvements in any of the clinical endpoints.

Limited case evidence suggests that LDN may also be effective in controlling symptoms of complex regional pain syndrome (CRPS) [38], a disease that often shows evidence of both local and low-level systemic inflammation [39]. Larger trials are needed to follow up on the one available case series report. Overall, while the literature is quite small, there is a consistent theme of LDN efficacy in controlling diseases with inflammatory components.

## An alternate explanation of LDN mechanism

While we believe much data is consistent with that claim that LDN works via novel anti-inflammatory channels, there are alternative compelling explanatory models of the LDN mechanism. The most prevalent hypothesis, advanced by Dr. Ian Zagon and colleagues, states that inducing a small and transient opioid blockade will prompt the body to compensate by upregulating both endogenous opioids and opioid receptors [40]. The opioid upregulation effect of temporary naltrexone or naloxone blockade has been demonstrated multiple times previously [41, 42]. This “opioid rebound” effect could have multiple impacts on health and quality of life, including enhanced endogenous analgesia and repression of critical immune factors [40].

Further research is needed with naltrexone and naloxone stereoisomers to determine the true mechanism of clinical action. In the meantime, we note that both the TLR4 and opioid receptor mechanisms may play a role in LDN action, as the hypotheses are not mutually exclusive.

## Why a low dosage?

Successful treatment of chronic pain with naltrexone may require low dosages. Theoretically, a complete blockade of endogenous opioid systems would not be a desirable outcome with a chronic pain patient. Basic science evidence supports that concept by showing that low- and high-dose opioid antagonists have quite different impacts on the physiologic system [43].

It may initially seem strange that a medication can have an opposite effect when given at a low dosage. However, there is a strong precedent for this concept—and with opioid-related drugs in particular. A paradoxical hyperalgesic effect of low-dose morphine was first widely reported in 1987 [44]. Morphine was administered via the IV route to rats after arthritis was induced using Freund’s adjuvant. A dose of 100 μg/kg produced clear analgesia, 50 μg/kg produced less significant analgesia, and 30 μg/kg showed no difference from saline. At around 10 μg/kg, however, the researchers saw the development of morphine *hyperalgesia*, which became most pronounced at 6 μg/kg. This finding, which has been replicated several times (e.g., [45]), suggests that there is a small window at which opioid analgesics produce the opposite effects than those typically expected. The dosage of morphine that appears to cause paradoxical hyperalgesia is approximately 1/10th of the dosage typically used to produce analgesia. We note that the dosage of naltrexone that is used to reduce pain is also approximately 1/10th of the dosage used for substance abuse treatment.

## Use of LDN in research studies

It is important to note that there are currently no guidelines for the clinical use of LDN. There is no FDA-approved use for naltrexone at any dosage for the treatment of chronic pain and inflammatory diseases. There is also no FDA-approved use of LDN for the treatment of any medical condition. Researchers using LDN must do so under an FDA Investigational New Drug (IND) application. While physicians have developed varying strategies for the use of LDN, none have been empirically validated. Therefore, in this section, we cover the use of LDN in published research trials and do not intend this discussion to be viewed as guidelines for the clinical use of LDN.

The typical dosage of LDN in published research is 4.5 mg. The medication is commonly given approximately an hour before bedtime, though some individuals reporting insomnia as a side effect are moved to a morning dosing. Individuals with side effects also have their dosage reduced to 3.0 mg. At the time of writing, naltrexone is commercially available only in a 50-mg tablet form, although one US-based company appears to be gathering regulatory approvals to market the 4.5 mg formulation. Because there is no commercial formulation of LDN, research studies obtain the medication via compounding pharmacies. Standard gelatin capsules and microcrystalline cellulose filler are commonly used.

In our research studies, the initial clinical benefits specific to LDN were difficult to distinguish from transient placebo effects. Separation from placebo may not be observed until at least 1 month after initiating treatment, with 2 months generally needed to obtain an estimate of efficacy.

There are no reports of LDN interactions with other medications. However, the sample sizes in studies have been very small, and there are undoubtedly a large number of interactions that have not been tested. Pharmacologically, there is little to expect in the way of interactions, though synergistic effects with anti-inflammatories and disease modifying antirheumatic drugs should be investigated. An obvious exception is LDN co-administered with an opioid analgesic. The most common question we receive about LDN is whether it can be given with opioid analgesics. It is possible that even a low dosage of naltrexone could cause a sufficient blockade of opioid receptors to reduce the effectiveness of opioid analgesics. In our studies, we excluded all individuals taking opioid analgesics. While there are published human data regarding ultralow-dose naltrexone co-administered with opioid analgesics [2, 3], we are not aware of the existence of co-administration studies using naltrexone in the LDN dosage range. Future studies may investigate the concomitant use of LDN and opioid analgesics—as it will likely be a commonly requested combination.

## Advantages of LDN

Because LDN is still an experimental therapy for chronic pain, there must be significant promise to justify recommending its use. LDN carries several advantages that may make it an attractive treatment option, which are reviewed below.

### Low cost

As a generic medication, naltrexone HCl is inexpensive. While pricing can vary considerably by region and pharmacy, the monthly cost of LDN appears to average US$35 per month. That cost includes compounding and assumes no insurance coverage. The price is lower than what would be paid for current on-patent medications for fibromyalgia, which can cost over US$100 per month.

### Low side effects

One of the most exciting aspects of LDN is the low reported incidence of adverse side effects. We have not seen incidences of ulcers, renal insufficiency, interference with warfarin and other common medications, increased heart attack or clotting risk, or other problems that can be seen with nonsteroidal anti-inflammatory drugs. We have observed no cases of severe adverse events in our research, and none have been reported from other laboratories. We have observed no withdrawal symptoms when LDN treatment is stopped, and withdrawal is not a known effect of treatment discontinuance [46]. However, the complete sample size of all LDN trials combined is still quite small and thus clinically useful data and experience are limited.

Side effects of LDN treatment are mild. In our research, participants have rated LDN as slightly more tolerable than placebo (91.0 versus 89.5 %, not significant). The most common side effect we have observed is the reporting of more vivid dreams, which is seen in approximately 37 % of the participants. In a minority of cases, patients report nightmares. As a side effect, vivid dreams develop rapidly (as soon as the first dosing) and decrease over time. It is unclear what mechanism may drive increased vividness of dreams. Individuals generally self-report increased effectiveness of sleep, so it is unlikely that the vivid dreams represent an adverse disruption of normal sleep patterns. It is important to note that increased vividness of dreams is also the most commonly reported side effect during placebo administration, so some cases may be driven by expectancy.

The frequency of headaches when taking LDN was slightly higher than during placebo administration, though more participants will need to be assessed in order to determine the statistical significance of the difference. Spontaneous headaches are common in individuals with fibromyalgia and frequently appeared in all stages of the clinical trials.

While not observed in research studies, some physicians have anecdotally reported anxiety and tachycardia as adverse reactions to LDN. As anxiety is a known symptom of opioid withdrawal, it is possible that some individuals would experience anxiety due to blockade of endogenous opioids. Further observation will need to be carried out to determine how common this adverse event is and how to best manage it.

For individuals without severe hepatic disease, there does not appear to be any need to frequently monitor hepatic function. Even at much larger dosages, naltrexone does not significantly change hepatic enzyme activity [47]. We have not observed any toxicity issues with chronic use.

### No known abuse potential

As an opioid antagonist, naltrexone is used as a treatment for substance abuse. LDN does not exert any euphoric or reinforcing effects, and we have observed no cases of LDN misuse or abuse. Furthermore, we have not seen the development of dependence and tolerance with the medication. In our studies, the cessation of LDN is generally followed by a slow return of symptoms to baseline levels.

## Disadvantages of LDN

As an off-label and experimental medication for pain, LDN does carry disadvantages. These disadvantages will now be discussed.

### Patients creating their own dosages

At the time of writing, LDN is not available at the 4.5-mg dosage that would be typical for the management of chronic pain. As such, many individuals may try to create their own dosing by subdividing 50-mg tablets. Internet resources that explain the process of splitting 50-mg tablets or creating a solution and dividing out liquid doses have been found. Such approaches will likely lead to unintended variability in the day-to-day dosing. The harm of such inconsistency is mitigated by the fact that it is very unlikely that someone could dangerously overdose on naltrexone. Still, patients taking responsibility for creating doses is far from optimal.

### Lack of proper dosage-finding experiments

It is highly probable that 4.5 mg is not the optimal dosage for all individuals with fibromyalgia, as it is rare for any pharmaceutical to have a one-size-fits-all dosage. In addition to obvious variables such as body mass index, individuals may differ in their metabolism, opioid receptor sensitivity, or microglia sensitivity to LDN. It is plausible that individuals who do not respond to 4.5 mg daily may respond to either lower or higher dosages. Other dosing schedules, such as twice a day, have not been explored in clinical studies. For now, the once daily 4.5-mg dosing schedule appears to be used without much critical analysis, as there are no published reports of even basic dose-ranging in human participants. Proper dosing studies need to be performed to determine the therapeutic range of the drug and to identify a process for determining an individual’s optimal dosage. The importance of determining proper dosing strategies is highlighted by animal research that suggests, for example, that while LDN may suppress tumors when used in the typical fashion, it may actually enhance tumor growth when administered more frequently [48].

### No hard data on long-term safety

Even though naltrexone has a long history of safe use with a wide range of large dosages, we know very little about the long-term safety of the drug when used chronically in low dosages. The low dosage is often cited as a reason for clinicians and patients to not be concerned about safety. However, we must be open to the possibility that the unique clinical effects possible with the low dosage could also present new health risks. There are no reported serious concerns to date. While inhibition of immune system parameters could theoretically raise the risk of infections or cancer due to decreased immunosurveillance, there have been no reports of such a side effect at any dosage of naltrexone.

### Not recognized by insurance companies

As an off-label, nonmainstream treatment, LDN may not be covered by insurance plans. As noted previously, the low overall cost of LDN may make it accessible even to patients who do not have it covered by insurance. Still, there will undoubtedly be a significant number of individuals who will find even a monthly cost of approximately US$35 to be prohibitively expensive. Therefore, the potential lack of insurance coverage is a downside of LDN.

## Beyond LDN

As a glial cell modulator, LDN is considered a medication of convenience. Naltrexone was not created as a microglia modulator. It is unlikely, therefore, that LDN represents the full promise of glial cell modulation in the treatment of chronic pain and inflammatory conditions. We now discuss some of the most promising compounds that may be tested in the near future.

### Dextro-naltrexone

While commercially available naltrexone is usually referred to simply as naltrexone HCl, it is actually the levo (also known as “left” or “−“) enantiomer of naltrexone. The levo form of naltrexone carries largely opioid antagonistic effects. The dextro (“right” or “+”) form of naltrexone was likely jettisoned during development because there was no known anti-abuse properties of the enantiomer.

Dextro-naltrexone, however, may be far more interesting in terms of anti-inflammatory and microglia-modulating properties. Preliminary data in animal models have already suggested that dextro-naltrexone may have a role in reducing pain and inflammation [22]. Not only does it appear to potently suppress microglia but it also exerts little activity on opioid receptors, which could translate into reduced risk of side effects related to systemic opioid blockade. Therefore, dextro-naltrexone might be administered at higher dosages, yielding greater microglia-suppressing activities while minimizing side effects. It is also possible that dextro-naltrexone, co-administered with opioid analgesics, might allow patients to realize the full benefits of opioid analgesia while simultaneously blocking many of the adverse effects.

Currently, dextro-naltrexone is not available for human use, and we are not aware of any studies of the compound tested in human subjects. There is also no source for obtaining dextro-naltrexone for human consumption. Getting dextro-naltrexone to clinical trials would require a great deal of time and money to navigate the necessary FDA and other regulations to ensure patient safety. It is unclear if any groups are progressing on this front (though the medication is referenced in a 2013 US patent application filing—US 13/799,287). We suggest that this line of research be adopted and dextro-naltrexone be tested on at least a small group of chronic pain patients to look at potential applications.

### Other compounds

LDN is not unique in receiving FDA approval for one purpose and then subsequently being discovered to also act as a glial cell modulator. Such compounds being tested in clinical trials include compounds such as minocycline [49] and dextromethorphan [50]. Further research will likely discover other compounds to have glial cell modulating properties, and opioid antagonists similar to naltrexone, such as nalmefene [51], may be good targets for further study.

Many other agents are currently being tested in animal models, such as fluorocitrate and 3-hydroxymorphinan, and it is likely that compounds are now being developed specifically for their TLR4-modulating properties. Other Toll-like targets are of interest as well, such as TLR-7 and TLR-9 blockage by hydroxychloroquine, which has been used successfully in inflammatory disorders such as systemic lupus erythematosus [52] and post-Lyme’s arthritis [53]. We expect glial cells modulators to be a central theme in future drug development efforts.

The potential of agents to suppress microglia extends beyond existing pharmaceuticals and includes botanicals. Several botanicals, such as stinging nettle, reishi mushroom, and curcumin, possess many key characteristics of potent glial cell modulators [54]. Most of these compounds and extracts are currently available for human use as supplements. However, research in this area has been confined to in vitro and animal in vivo work. Future clinical trials may test several of these botanicals for treating fibromyalgia and other conditions.

## Public opinion

LDN has garnered a public reputation that is not commonly seen with pharmaceutical treatments. There is a considerable grassroots effort in the UK to have the medication recognized by the National Health Service (NHS). In the USA, a book has been published specifically on LDN [55]. Considerable information and misinformation is disseminated via the internet. Some sources recommend LDN for a vast range of medical conditions, the majority of which have not been subjected to any scientific study. To date, we are aware of initial clinical evidence of efficacy only in fibromyalgia, Crohn’s disease, multiple sclerosis, and complex regional pain syndrome. Whatever uses LDN may ultimately be found to have, it is clear that there is presently a great gap between the claims and the scientific evidence.

Individuals who are normally resistant to consuming mainstream pharmaceuticals are nonetheless often willing to trial LDN. Patient affinity for LDN may be driven somewhat by the “low-dose” preface, which can be agreeable to individuals who have experienced negative side effects from other medications. Clinicians prescribing LDN should be aware that many patients will come into the treatment with considerable expectations which could drive placebo effects. Patients may even approach a clinician with a specific request to be prescribed LDN.

## Next steps

Throughout this review, we have raised several suggestions for future research projects, including dose ranging studies for LDN, development and clinical testing of dextro-naltrexone, clinical testing of other available microglia modulators, and clinical trials of LDN administered concomitantly with opioid analgesics. We will now highlight a few additional research directions:

### Test LDN in other inflammatory conditions

Our findings that baseline ESR may be associated with LDN response suggest that other inflammatory conditions, such as rheumatoid arthritis, polymyalgia rheumatica, and lupus, may benefit from LDN. LDN may serve as a concomitant medication when immunomodulatory therapies are not effective or not well tolerated by the patient. We propose that pilot trials could be carried out to test LDN in inflammatory and autoimmune conditions.

### Better tracking of inflammatory markers during LDN treatment

Future studies examining LDN should determine if positive response is associated with a reduction of ESR or other measures of inflammation (high sensitivity C-reactive protein, secreted cytokines in blood plasma, growth factors, matrix metalloproteinases, etc.). Such information would help provide mechanistic information about the treatment. Examination of intracellular immune processes may further shed light on the mechanism of LDN treatment. Measurements of immune activity should be collected at least immediately before and after treatment or when the primary outcomes are assessed.

## Conclusions

The totality of the basic and clinical research to date suggests that LDN is a promising treatment approach for chronic pain conditions thought to involve inflammatory processes. The clinical data supporting its use are very preliminary, and more research is needed before the treatment approach can be widely recommended. Critical parameters such as dosing still need to be refined. LDN may emerge as the first of many glial cell modulators that could be used to treat chronic conditions, with more specifically targeted medications developed in the future. As conventional anti-inflammatories have poor blood brain-barrier permeability, we expect centrally active immune modulators to be an area of interest in the future.
